# Automated radiosynthesis and clinical experience of [^18^F]SMBT-1 PET imaging for *in vivo* evaluation of reactive astrocyte in Parkinson's disease: a pilot study

**DOI:** 10.3389/fnume.2025.1718255

**Published:** 2025-12-08

**Authors:** Peerapon Kiatkittikul, Pradith Lerdsirisuk, Laksika Bhuthathorn, Arthita Choolam, Attapon Jantarato, Pattanapong Kongsakorn, Wisakha Chintawan, Thanapon Sringam, Nirawan Fonghoi, Saiphet Vanprom, Ryuichi Harada, Nobuyuki Okamura, Shozo Furumoto, Chetsadaporn Promteangtrong

**Affiliations:** 1 National Cyclotron and PET Centre, Chulabhorn Hospital, Chulabhorn Royal Academy, Bangkok, Thailand; 2 Department of Medicine, Chulabhorn Hospital, Chulabhorn Royal Academy, Bangkok, Thailand; 3 Department of Pharmacology, Faculty of Medicine, Tohoku Medical and Pharmaceutical University, Sendai, Japan; 4 Research Center for Accelerator and Radioisotope Science, Tohoku University, Sendai, Japan

**Keywords:** [^18^F]SMBT-1, MAO-B, reactive astrocyte, neuroinflammation, PET, Parkinson’s disease

## Abstract

**Background:**

Neuroinflammation plays an important role in progression of Parkinson disease (PD). [^18^F]SMBT-1 is a promising novel radiotracer for *in vivo* evaluation of reactive astrogliosis.

**Methods:**

The automated radiosynthesis of [^18^F]SMBT-1 was optimized and performed production by using the Synthra RNplus synthesizer module, and the quality of the labeled tracer was evaluated. Total 5 participants, 2 PD and 3 Healthy Control were enrolled. Cognitive assessments were performed in all participants while H&Y scales and MDS-UPDRS were performed only in PD. All participants underwent [^18^F]SMBT-1 PET/MRI and [^18^F]FDOPA PET/CT within 2-week intervals. Demographic and imaging data were collected. The correlation between [^18^F]FDOPA uptake and [^18^F]SMBT-1 uptake was analyzed by Spearman's correlation.

**Results:**

[^18^F]SMBT-1 was successfully synthesized via nucleophilic substitution of a tosylate precursor, followed by a deprotection step. After purification and formulation, [^18^F]SMBT-1 was obtained with an average decay-corrected radiochemical yield of 36.56 ± 11.55% and molar activity as 396 Gbq/*μ*mol at the end of synthesis (*n* = 7). Moderate PD defined by the [^18^F]FDOPA showed increased [^18^F]SMBT-1 in prefrontal cortex, temporal lobes, striatum, thalamus, pons, medullar, and midbrain while severe PD showed globally increased [^1^⁸F]SMBT-1. Healthy control also showed globally increased [^18^F]SMBT-1 uptake. There was no significant correlation between the degree of [^18^F]FDOPA uptake and [^18^F]SMBT-1 uptake in any brain region.

**Conclusion:**

The automated radiosynthesis of [^18^F]SMBT-1 are suitable for routine production without any immediate complication reported after administration. Moderate PD shows decreased astrocyte function as they lose neuroprotective astrocytes while severe PD shows increased astrocyte function as increased neurotoxic astrocytes.

## Introduction

Parkinson's disease (PD) is the second most common neurodegenerative disorder, with increasing incidence and prevalence over the years (1). The pathological hallmark of Parkinsons’ disease is the progressive degeneration of dopaminergic neurons in the substantia nigra pars compacta (2). Although the etiology of Parkinson's disease remains unclear, current evidence suggests that abnormal alpha-synuclein protein accumulation and neuroinflammation processes associated with progressive dopaminergic neuronal loss plays an important role in disease progression (3, 4).

Astrocytes are the most abundant glial cells in the brain. They play a crucial role in maintaining brain homeostasis, regulating synaptic activity, supporting neuronal metabolism, and also play a part in neuroinflammatory processes (5). When injured, astrocytes can change into reactive astrocytes, which can be divided into two main subtypes; neurotoxic (A1) subtype which can release neurotoxin, resulting in more inflammation and neuronal death, and the neuroprotective (A2) subtype which promotes neuronal repair and recovery (6). Monoamine oxidase (MAO) are enzymes which plays a role in neurotransmitter metabolism, having two forms: MAO-A and MAO-B. Importantly, MAO-B is highly expressed in immune cells, especially in reactive astrocytes, and can serve as a surrogate marker for reactive astrogliosis (7, 8).

Positron emission tomography (PET) has emerged as a tool for *in vivo* evaluation of neuroinflammation by using specific radiotracers targeting ligands such as I_2_BS or MAO-B (9). Earlier PET tracers targeting the MAO-B enzyme, such as [^11^C]L-deprenyl and [^11^C]L-deprenyl-D_2_, showed very good binding affinity and selectivity to the MAO-B. However, there are limitations to these radiotracers as they irreversibly bind onto MAO-B which limits repeated PET imaging and quantification analysis, they are labelled with carbon-11 with very short half-life and the need for an on-site cyclotron, and metabolism of these tracers produces methamphetamine as a byproduct (9, 10).

To overcome these limitations, newer tracers such as [^18^F]SMBT-1 were developed. [^18^F]SMBT-1 demonstrates high selectivity for MAO-B with a reversible binding property, can be labelled with F-18, and can be completely metabolized without leaving any byproducts (11, 12). To date, [^18^F]SMBT-1 has been validated as a safe and promising radiotracer for *in vivo* evaluation of reactive astrocytes and has already been used in Alzheimer's disease and its spectrum (13, 14). However, no study has yet applied it for the *in vivo* evaluation of Parkinson's disease. In this study, we aim to optimize the radiosynthesis method from manual to automated procedure to maintain high reproducibility, optimal radioactivity yield, and radiochemical purity and explored the reactive astrocyte function in Parkinson's disease using [^18^F]SMBT-1 as compared to the healthy control.

## Materials and methods

### Radiosynthesis of [^18^F]SMBT-1

**Figure 1:**
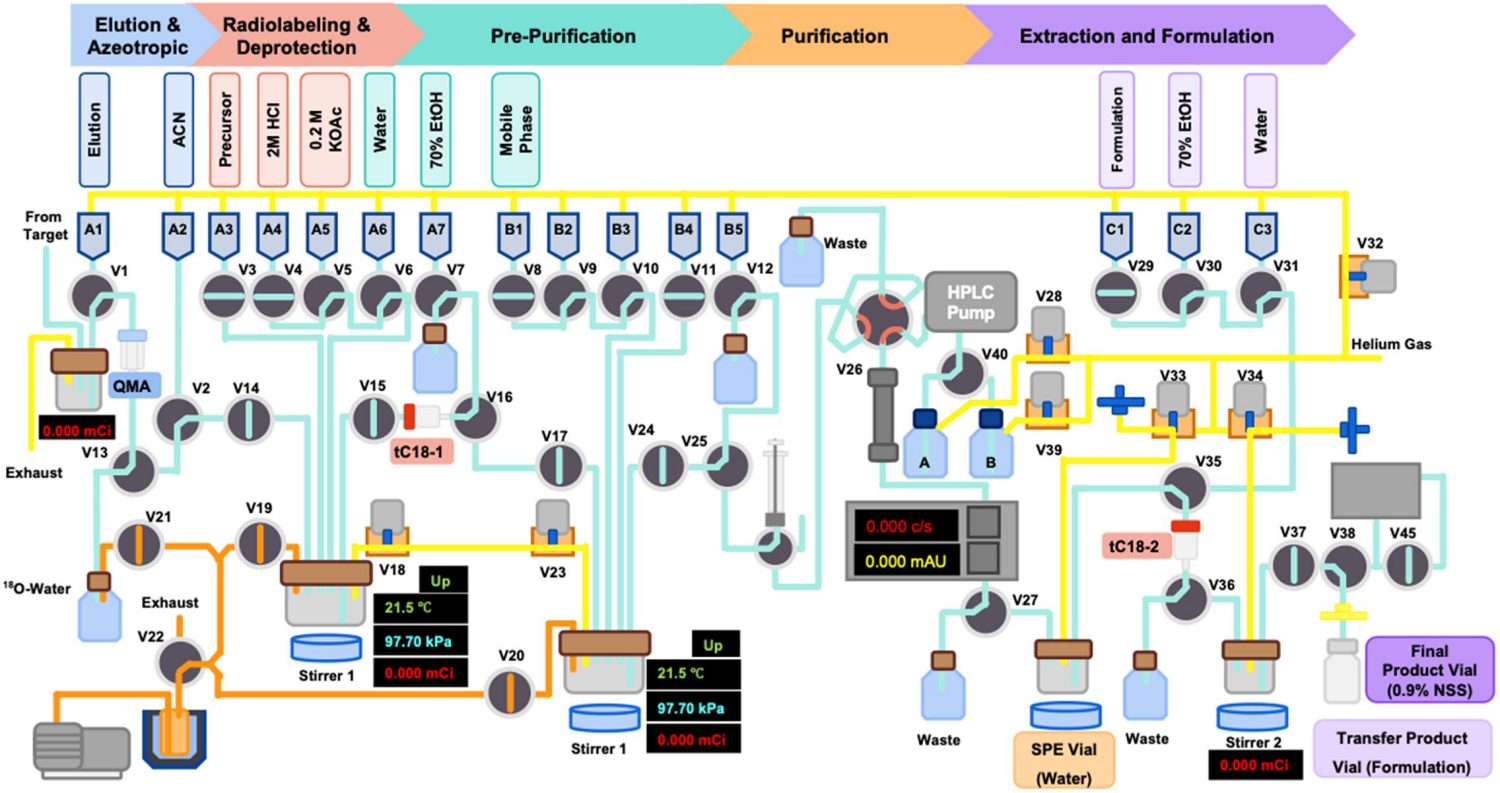
Adapt from graphic user interface of synthra RNplus synthesizer module and synthesis preparation for [^18^F]SMBT-1 radiosynthesis.

**Scheme 1:**
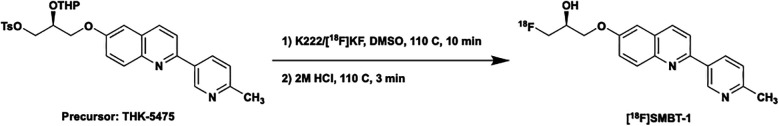
Radiosynthesis scheme of [^18^F]SMBT-1.

**Figure 2:**
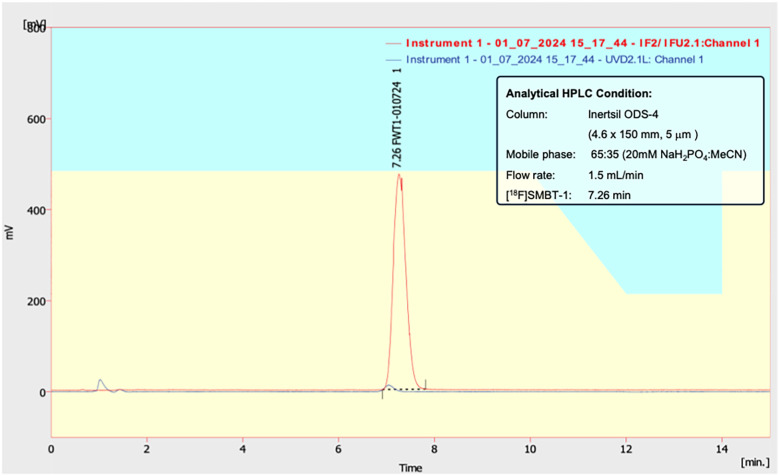
The radiochromatogram of [^18^F]SMBT-1 product on the analytical HPLC.

Automated synthesis of [^18^F]SMBT-1 was performed using the Synthra RNplus synthesizer module (Synthra GmbH, Germany). The synthesis preparation and setup on the Synthra RNplus synthesizer module were shown in Figure 1. The chemical structure and radiosynthesis scheme is shown in Scheme 1. [^18^F]Fluoride was produced via the ^18^O(p,n)^18^F nuclear reaction on enriched [^18^O]H₂O using a PETtrace 800 cyclotron (GE Healthcare). The irradiated target solution was transferred to a Sep-Pak Light Accell Plus QMA cartridge (Waters), pre-conditioned with potassium carbonate (K_2_CO_3_) followed by water. Trapped [^18^F]fluoride was eluted with 1.0 mL of a solution containing Kryptofix®222 (17.8 mg/mL in acetonitrile) and K_2_CO_3_ (11.5 mg/mL in water). The eluted solution underwent azeotropic drying under helium flow at 110°C with magnetic stirring. Following complete evaporation, 2.0 mg of the tosylate precursor,(S)-(2-methylpyrid-5-yl)-6-{[2-(tetrahydro-2H-pyran-2-yloxy)-3-tosyloxy] propoxy}quinoline (THK-5475, Hebei Sundia MediTech, China), dissolved in 0.9 mL of dimethyl sulfoxide (DMSO), was added and heated at 110°C for 10 min. To deprotect the hydroxy group, 0.2 mL of 2 M hydrochloric acid (HCl) was added to the reaction mixture, which was then stirred for an additional 3 min at 110°C. The reaction was quenched by addition of 8.0 mL of 0.2 M potassium acetate (KOAc) aqueous solution. The crude mixture was subjected to solid-phase extraction using a pre-activated Sep-Pak tC18 Plus cartridge (Waters), washed with 10.0 mL of water, and eluted with 1.2 mL of 70% ethanol (EtOH) into Reactor 2. The eluted product was diluted with 2.8 mL of mobile phase [20 mM Sodium dihydrogen phosphate (NaH_2_PO_4_)/acetonitrile (ACN), 62:38, v/v] and transferred to the injector of a semipreparative high-performance liquid chromatography (HPLC) system. Purification was carried out using an Inertsil ODS-4 column (10 × 250 mm, 5 μm, GL Sciences Inc.) under isocratic conditions with the same mobile phase at a flow rate of 5.0 mL/min, product peak should be found around 16–17 min after injection. The collected peak of [^18^F]SMBT-1 was diluted with sterile water (45.0 mL) and then load onto tC18 cartridge-2, Then rinsed the SPE with sterile water 15.0 mL, then performed the extraction and formulation and then transfered to the final product pass through the sterile membrane filter 0.22 μm. [^18^F]SMBT-1 was obtained in radiochemical purity >95% as confirmed by analytical HPLC (4.6 × 150 mm, 5 μm, Inertsil ODS-4 column [GL Sciences Inc.]; 20 mM NaH_2_PO_4_/ACN, 65:35, v/v, flow rate = 1.5 mL/min). The radiochromatogram of [^18^F]SMBT-1 product on the analytical HPLC was shown in Figure 2. Detailed synthesis parameters, the optimization results and the conditions of semi-preparative and analytical HPLC are provided in Supplementary Information.

### Radiosynthesis of [^18^F]FDOPA

[^18^F]FDOPA was produced routinely via automated nucleophilic synthesis using the NEPTIS® Perform synthesizer module (ORA, Belgium) equipped with a commercially available cassette-based chemistry setup (ABX, Germany). The synthesis followed standard protocol (15). Radiochemical purity was >95%, as confirmed by analytical HPLC (4.6 × 150 mm, 5 μm, Atlantis T3, Water) under isocratic conditions.

### Study protocol

This study prospectively recruited 7 participants, including 4 patients with Parkinson's disease and 3 healthy controls. Patients with Parkinson's disease were diagnosed as either clinically established PD or clinically probable PD according to the Movement Disorder Society (MDS) clinical diagnostic criteria, confirmed by a neurologist. Eligible PD participants were required to be cognitively normal. Healthy controls were required to be around 50–70 years without any history or symptoms related to PD or other neurodegenerative disease. Participants with any history of stroke, epilepsy, psychiatric disorder, prior significant head trauma, malignancy, pregnancy or lactation, or PD patients who are currently receiving MAO-B inhibitors were excluded from this study.

This study was approved by the ethics committee of Chulabhorn Royal Academy. All participants were recruited through the neurologist, while healthy controls were recruited through public advertisement. All participants were informed about the study protocol and signed the consent form before recruited into this study. Disease severity in PD patients was assessed by the neurologist using both the Hoehn and Yahr (H&Y) scale and the MDS-UPDRS part III. The cognitive assessment with Montreal Cognitive Assessment (MOCA) scores was assessed in all participants, and the participants were further excluded from this study if they were cognitively abnormal. After passing the neurological assessment, each participant underwent two PET imaging within 2-week interval, including [^18^F]FDOPA PET/CT and [^18^F]SMBT-1 PET/MRI.

### [^18^F]SMBT-1 PET/MRI brain

PET/MRI images were acquired 60–90 min after intravenous administration of 166.5–203.5 MBq of [^18^F]SMBT-1. All data were collected on a 3.0-T hybrid PET/MRI scanner (Biograph mMR, Siemens Healthcare GmbH, Erlangen, Germany) using a single-bed list-mode PET acquisition performed simultaneously with MRI. A molecular head and neck coil was used, with attenuation correction applied for the coil.

For magnetic resonance–based attenuation correction (MRAC), a high-resolution DIXON sequence was obtained (voxel size, 1.3 × 1.3 × 2.0 mm; TE, 1.28/2.51 ms; TR, 4.14 ms). Postprocessing employed five-compartment segmentation (air, fat, soft tissue, lung adaptive, and bone).

The MRI protocol included the following sequences: sagittal 3D T1-weighted MPRAGE for morphometric analysis; axial time-of-flight (TOF) angiography of the brain; sagittal T2-weighted FLAIR with fat suppression; axial T2-weighted imaging with fat suppression; axial diffusion-weighted imaging (DWI); axial susceptibility-weighted imaging (SWI); coronal T2-weighted imaging; and coronal-oblique T1 inversion recovery (IR) focused on the hippocampus. The images were reviewed by a radiologist to exclude the presence of stroke and any structural brain abnormalities.

PET images were reconstructed using an ordered subset expectation maximization algorithm with point spread function modeling (OSEM + PSF, HD-PET). Reconstruction parameters included six iterations, 21 subsets, a 512 × 512 matrix, a zoom factor of 2, and an all-pass filter.

For data analysis, images were displayed in axial, coronal, and sagittal planes. Standardized uptake value ratios (SUVR) were generated using PMOD software (Bruker, Switzerland) for improved visualization. PET images were automatically co-registered for each subject using the automatic voxel-of-interest (VOI) method and subsequently registered to individual T1-weighted MRI data. The T1-weighted MRI was used for both image registration and delineation of brain reference regions. All MRI data were standardized to the Montreal Neurological Institute (MNI) T1-weighted MRI template atlas.

VOIs were automatically outlined on the normalized MRI using the maximum probability approach based on the automated anatomical labeling (AAL) merged atlas. Regional SUVR values of [^18^F]SMBT-1 were calculated using the subcortical white matter as the reference region. The reference SUV was then applied to generate SUVR images by function-scaling within PMOD.

All images were visually assessed by two experienced nuclear medicine physicians using Syngo.Via DICOM Viewer (Siemens Healthcare GmbH, Erlangen, Germany). Abnormally increased radiotracer uptake within the brain parenchyma was considered a positive finding.

### [^18^F]FDOPA PET/CT brain

[^18^F]FDOPA PET/CT images were acquired approximately 90–100 min after intravenous administration of 185 MBq of [^18^F]FDOPA on a 128-slice Biograph Vision Edge 600 PET/CT scanner (Siemens Healthcare GmbH, Erlangen, Germany).

For data analysis, images were displayed in axial planes. Visual interpretation was performed according to established grading criteria: Normal—symmetrical crescent-shaped uptake in both putamen and caudate, mild abnormal—normal uptake on one side with reduced uptake contralaterally, particularly in the posterior putamen, moderate abnormal—bilateral reduction of uptake in the putamen, and severe abnormal—bilateral reduction of uptake in both putamen and caudate.

For semiquantitative analysis, PET data were processed using the Syngo.Via Striatal-to-Occipital Ratio (SOR) application. PET images were co-registered to the FDOPA Syngo.Via template, after which VOIs were automatically generated for both the caudate nuclei and putamina. SUV values from these striatal regions were divided by the SUV of the occipital cortex to obtain SUVRs. Individual SUVRs were then compared against the normal database provided within the Syngo.Via software.

### Statistical analysis

All demographic data were reported in numbers and percentages. Pearson's correlation was used to evaluate the correlation between dopaminergic neuron function with [^18^F]FDOPA and the degree of reactive astrocyte activity by [^18^F]SMBT-1. The statistical analysis was conducted using IBM SPSS Statistics for Windows, version 28.0 (IBM Corp., Armonk, New York, United States).

## Results

### Radiosynthesis of [^18^F]SMBT-1

[^18^F]SMBT-1 was synthesized by nucleophilic substitution of a tosylate precursor (THK5475), follow by a deprotection step. After purification with semi-preparative HPLC, extraction and formulation, [^18^F]SMBT-1 was obtained with 36.56% ± 11.55% as the average decay-corrected radiochemical yield at end of synthesis. The average molar activity was 396 GBq/μmol, with a range of 15–2,242 GBq/μmol. The radiochemical yield of 7 batches of [^18^F]SMBT-1 production are summarized in the Table 1.

**Table 1:** Radiochemical yield of [^18^F]SMBT-1 in 7 batches of production on synthra RNpuls synthesizer module.

No.	Received F-18 (GBq)	[^18^F]SMBT-1 at EOS (GBq)	EOS yield (%)	Decay corrected yield (%)
1	118.4	20.20	17.06	28.81
2	122.1	25.42	20.82	35.15
3	151.7	24.46	16.12	27.22
4	110.0	34.67	31.52	52.21
5	108.0	31.89	29.54	51.80
6	131.9	32.67	24.77	37.33
7	82.8	11.54	13.93	23.38

### Radiosynthesis of [^18^F]FDOPA

The routine radiosynthesis of [^18^F]FDOPA was performed base-on the commercially cassette-based production by nucleophilic substitution via the NEPTIS® Perform synthesizer module. The decay corrected yield of [^18^F]FDOPA (*n* = 26) were 9.60% ± 3.61%.

### Clinical study

Seven participants were enrolled in this study. Two patients with Parkinsons’ disease (PD) were subsequently excluded due to presence of cognitive abnormalities detected during the neurocognitive assessment performed prior to PET imaging. Thus, a total of five participants were recruited in the final study cohort, comprised of two Parkinson's disease patients and three healthy controls (HC), as shown in Table 2. All participants successfully underwent both [^18^F]SMBT-1 PET/MRI and [^18^F]FDOPA PET/CT without any immediate adverse event after the radiotracer administration.

**Table 2:** Demographic data.

No.	Group	Sex	Age	Education level	Underlying disease	MOCA	H&Y scale	MDS-UPDRS part III				
								Tremor	Rigidity	Bradykinesia	Axial symptom	Total score
1	Parkinson's disease	Male	50	Bachelor's degree	No	28	2	9	5	10	2	26
2	Parkinson's disease	Female	60	High school	Hypertension, Dyslipidemia	25	3	6	4	13	11	34
3	Healthy	Male	66	High school	Dyslipidemia, HCV infection	25	-	-	-	-	-	-
4	Healthy	Female	65	Bachelor's degree	No	29	–	–	–	–	–	–
5	Healthy	Female	69	Bachelor's degree	Dyslipidemia	26	–	–	–	–	–	–

All three healthy controls demonstrated normal [^18^F]FDOPA uptake, confirming the absence of underlying dopaminergic neuron deficit and excludes any potential confounding effect from Parkinsonism. In contrast, two PD participants demonstrated abnormalities in [^18^F]FDOPA, with case 1 exhibiting a moderate dopaminergic neuron deficit and case 2 exhibiting a severe dopaminergic neuron deficit.

**Figure 3:**
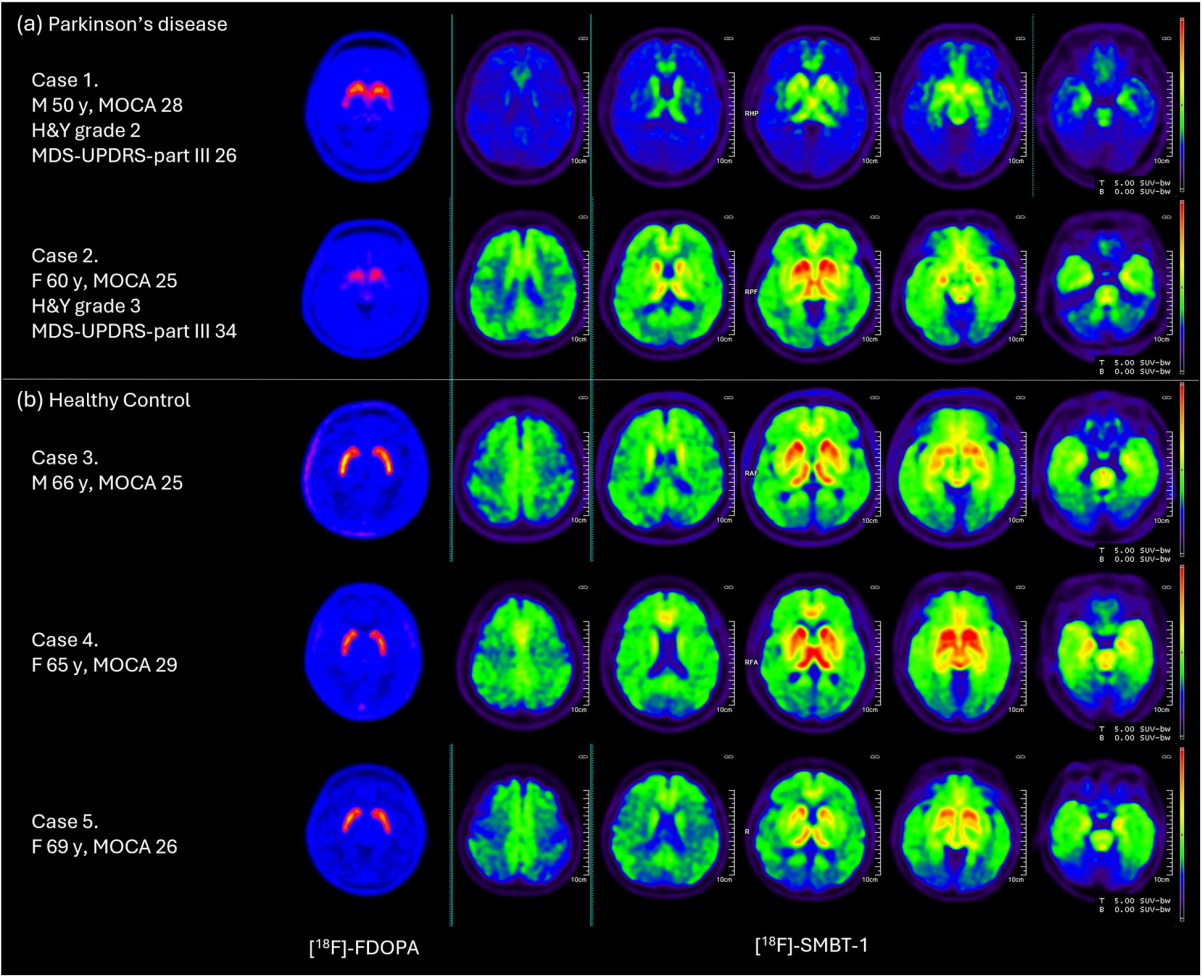
Demonstrate all cases enrolled in this study. A). Parkinson's disease patients in this study. Case 1, a 50-year-old man with H&Y scale of 2 and MDS-UPDRS part III score of 26 demonstrated moderate [^18^F]FDOPA abnormalities. [^18^F]SMBT-1 uptake only increased in the prefrontal cortex, temporal lobes, striatum, thalamus, pons, medulla, and midbrain. Case 2, a 60-year-old woman with H&Y scale of 3 and MDS-UPDRS part III score of 34 demonstrated severely [^18^F]FDOPA abnormalities. [^18^F]SMBT-1 uptake increased in multiple areas in both cortical and subcortical brain regions. B). Healthy control (HC) in this study all cases in this group show normal [^18^F]FDOPA uptake. [^18^F]SMBT-1 show increased uptake in multiple areas in both cortical and subcortical brain regions.

In the healthy control group, [^18^F]SMBT-1 uptake was increased across multiple cortical and subcortical regions, with the highest uptake in the striatum, as shown in Figure 3A. In Case 1 of the Parkinson's disease group, [^18^F]SMBT-1 showed increased radiotracer uptake in the prefrontal cortex, temporal lobes, striatum, thalamus, pons, medulla, and midbrain with moderate dopaminergic deficit. However, in Case 2 of the Parkinson's disease group,[^18^F]SMBT-1 showed globally increased radiotracer uptake in multiple brain areas with a severe dopaminergic deficit defined by [^18^F]FDOPA as shown in Figure 3B. For evaluation of the relationship between the dopaminergic function and reactive astrocyte activity, there was no statistically significant correlation between the degree of [^18^F]FDOPA uptake and [^18^F]SMBT-1 uptake across multiple brain regions as shown in Table 3.

**Table 3:** Spearman's correlation between the degree of [^18^F]FDOPA and [^18^F]SMBT-1.

SUVR [^18^F]SMBT-1	SUVR [^18^F]FDOPA			
	Caudate		Putamen	
	Spearman's correlation	*p*-value	Spearman's correlation	*p*-value
Prefrontal	−0.2 (−0.926,0.841)	0.747	−0.7 (−0.980,0.508)	0.188
Orbitofrontal	−0.1 (−0.910,0.868)	0.873	−0.6 (−0.972,0.625)	0.285
Frontal	−0.2 (−0.926,0.841)	0.747	−0.7 (−0.980,0.508)	0.188
Insular	0.1 (−0.868,0.910)	0.873	−0.6 (−0.972,0.625)	0.285
Anterior cingulate gyrus	0.1 (−0.868,0.910)	0.873	−0.6 (−0.972,0.625)	0.285
Middle cingulate gyrus	−0.2 (−0.926,0.841)	0.747	−0.7 (−0.980,0.508)	0.188
Posterior cingulate gyrus	−0.2 (−0.926,0.841)	0.747	−0.7 (−0.980,0.508)	0.188
Hippocampus	0.1 (−0.868,0.910)	0.873	−0.6 (−0.972,0.625)	0.285
Parahippocampus	0.1 (−0.868,0.910)	0.873	−0.6 (−0.972,0.625)	0.285
Amygdala	−0.2 (−0.926,0.841)	0.747	−0.7 (−0.980,0.508)	0.188
Occipital	−0.1 (−0.910,0.868)	0.873	−0.6 (−0.972,0.625)	0.285
Lateral temporal	−0.2 (−0.926,0.841)	0.747	−0.7 (−0.980,0.508)	0.188
Superior temporal	0.1 (−0.868,0.910)	0.873	−0.6 (−0.972,0.625)	0.285
Inferior temporal	−0.154 (−0.919,0.854)	0.805	−0.667 (−0.977,0.552)	0.219
Parietal	−0.2 (−0.926,0.841)	0.747	−0.7 (−0.980,0.508)	0.188
Striatum	0.1 (−0.868,0.910)	0.873	−0.6 (−0.972,0.625)	0.285
Thalamus	0.1 (−0.868,0.910)	0.873	−0.6 (−0.972,0.625)	0.285
Pons	0.5 (−0.705,0.962)	0.391	−0.2 (0.926,0.841)	0.747
Medulla	0.1 (−0.868,0.910)	0.873	−0.6 (−0.972,0.625)	0.285
Midbrain	0.1 (−0.868,0.910)	0.873	−0.6 (−0.972,0.625)	0.285

## Discussion

Neuroinflammation plays an important role in disease progression in Parkinson's disease. MAO-B is highly expressed in the immune cell, especially in reactive astrocytes and can serve as a surrogate marker for reactive astrogliosis (7, 8). However, previous studies have reported that MAO-B expression increases with age (16). Therefore, we decided to compare the findings from PD patients with an age-matched healthy control in this study.

The radiosynthesis of [^18^F]SMBT-1 was performed using the Synthra RNplus synthesizer module, which is suitable for both routine production and research and development of novel ^18^F-labeled tracers. The synthesis procedure and time control file were adapted and optimized based on the manual radiosynthesis described in the preclinical evaluation of [^1^⁸F]SMBT-1 (11). Initially, the amounts of all chemical reagents were kept consistent with the manual method to evaluate the automated sequence as shown in Supplementary Table S1. This allowed testing of the gas and solution flow, heating steps, solution transfer timing, and the overall reaction performance. After the initial synthesis, the distribution of ^18^F-radioactivity remaining on various components of the module was measured and analyzed (Supplementary Table S2). The results indicated that a significant portion of radioactivity (55.16%) remained on the QMA cartridge, likely due to the small volume of the eluting solution (0.58 mL). Additionally, the tC18 cartridge-1 retained 20.16% of the radioactivity, suggesting that the volume of 70% ethanol (0.70 mL) used for eluting the crude product to reaction vial 2 was insufficient. Only a trace amount of final product was obtained (decay-corrected yield = 0.88%). This low yield was assumed to be due to incomplete deprotection and inadequate neutralization using 4.0 mL of 0.2 M KOAc. In the second synthesis trial, the volume of the eluting solution in A1 vial was increased to 1.0 mL, and the 70% ethanol in A7 vial was also increased to 1.0 mL, aiming to reduce residual activity on the QMA and tC18 cartridge-1. Additionally, the deprotection step duration was adjusted, and the volume of 0.2 M KOAc was doubled to 8.0 mL to improve the radiochemical yield. This optimization increased the decay-corrected yield to 16.09% and reduced the activity on the QMA cartridge to 6.06%. However, a high residual activity (35.62%) remained on the tC18 cartridge-1. Gas chromatography analysis also showed that the acetonitrile content exceeded acceptable limits. In the third optimization trial, to reduce both the residual activity on tC18 cartridge-1 and the acetonitrile content in the final product, the 70% ethanol volume in A7 vial was increased to 1.2 mL, and the water volume in the SPE vial was increased to 45 mL. This trial yielded favorable results: residual activity on tC18 cartridge-1 was reduced to 3.32%, and the final product was obtained with a decay-corrected yield around 14.2%.

All three synthesis trials guided the final confirmation and optimization of the time control file for the [^18^F]SMBT-1 radiosynthesis method. To maximize the recovery of radioactivity in the final product, the formulation solution volume was separated to 4.0 mL and loaded into the C1 vial to flush any residual activity retained in the tC18 cartridge-2 and the transfer line leading to the product vial that contained more formulation solution as 4.0 mL. Following this adjustment, the finalized synthesis conditions and parameters were established and are summarized in Supplementary Table S2. Method validation and full quality control (QC) were then carried out through three independent validation runs, including an evaluation of the final product's stability. The validated radiosynthesis method of [^18^F]SMBT-1 yielded impressive results, achieving an average decay-corrected radiochemical yield of 30.20% ± 4.32%, with radiochemical purity exceeding 98%. Furthermore, stability testing demonstrated that [^18^F]SMBT-1 maintained high radiochemical purity (>95%) for up to 10 h post-synthesis. The results of method validation and QC were shown in Table 4 and the chromatogram of the stability result was shown in Supplementary Figure S2.

**Table 4:** The method validation and quality control (QC) results.

Method validation of [^18^F]SMBT−1		Validation 1	Validation 2	Validation 3
Production	SOS: start activity of F-18	118.4 GBq	122.1 GBq	151.7 GBq
	EOS: final product activity	20.20 Gbq	25.42 GBq	24.46 GBq
	Synthesis time	83 min	83 min	83 min
	Decay corrected activity	34.11 GBq	42.29 GBq	41.29 GBq
	%Yield (decay corrected)	28.81%	35.16%	27.23%
Quality Control	Appearance	Colourless and clear	Colourless and clear	Colourless and clear
	pH	7.23	7.30	7.64
	Radionuclide Identity (Half-life)	108.341 min	110.960 min	111.026 min
	Radiochemical Purity (HPLC)	>99.9%	>99.9%	99.8%
	Radiochemical Identity (HPLC)	Rt STD: 6.86 min	Rt STD: 6.74 min	Rt STD: 7.03 min
		Rt sample: 7.10 min	Rt sample: 7.00 min	Rt sample: 6.91 min
		RRT: 1.03	RRT: 1.04	RRT: 0.98
	Solvent residue (GC)	ACN: 0.140 mg/mL	ACN: 0.0139 mg/mL	ACN: N/D
		DMSO: N/D	DMSO: N/D	DMSO: N/D
		EtOH: 4.94%	EtOH: 3.80%	EtOH: 4.29%
	Pyrogenicity (BET)	<10.0 EU/mL	<10.0 EU/mL	<10.0 EU/mL
	Filter integrity (Bubble test)	55 psi	51 psi	48 psi
	Sterility test	Sterile	Sterile	Sterile
	Stability (RCP >95%)	10 h	10 h	10 h

From our results, the healthy control group showed increased [^18^F]SMBT-1 uptake in multiple areas of both cortical and subcortical regions, with the highest uptake in the striatum. These results are consistent with the previously reported MAO-B distribution in the brain (16). The degree of [^18^F]SMBT-1 uptake in the healthy control group seems to be higher than previously reported; possibly because our population of healthy controls were age-matched with an age range between 50 and 70 years, consistent with prior reports that MAO-B expression increases with age. Additionally, about 20%–30% of older healthy controls have been shown to demonstrate significant amyloid deposition in the brain (17), which correlates with a previous study (14) that reported a higher abnormal amyloid protein deposition in the brain significantly correlates with the higher [^18^F]SMBT-1 uptake in the brain in healthy controls. However, we did not perform amyloid PET imaging to exclude the possibility of this confounding factor, which is one of the limitations of this study.

In our cohort of patients with Parkinson's disease, we found that patients with moderate dopaminergic deficit showed less [^18^F]SMBT-1 uptake as compared to the patient with severe dopaminergic deficit. This result was probably due to a decline in neuroprotective astrocyte function in moderate PD and subsequent increased neurotoxic astrocyte function in severe PD, corresponding to the hypothesis from previous studies. An *in vivo* study by Ballweg A, et al. (18) reported a globally increased reactive astrocyte activity in the brain using [^18^F]DED in patients with Parkinson's disease, most pronounced at the basal ganglia. Another study by Wilson H. et al. (19) reported a significant increase in [^11^C]BU99008 uptake in the brain of patients with early Parkinson's disease, a radiotracer reflecting neuroprotective astrocyte activity, with a decline in tracer uptake in patients with advanced disease, reflecting decreased neuroprotective astrocyte activity as the disease progresses.

A study reported an increase in reactive astrocytes, especially the neurotoxic subtype, in patients with advanced neurodegenerative disease (20), and a post-mortem study showed increased MAO-B expression in patients with Parkinson's disease (21). This may suggest that in very late stages of Parkinson's disease, there may be an increase in neurotoxic reactive astrocyte, resulting in increased MAO-B expression and inflammation.

However, given the very low number of participants in our study, further investigation with a larger number of participants, particularly patients with early Parkinson's disease, would provide greater insight support this hypothesis.

In addition, we explored the relationship between the degree of dopaminergic deficit, defined by the SUVR of [^18^F]FDOPA in the caudate and putamen, and the degree of reactive astrocyte activity, defined by the SUVR of [^18^F]SMBT-1 across multiple brain regions. No statistically significant correlations were observed in our study which was probably due to the small sample size. Further investigation with larger cohort may clarify whether a meaningful association exists between these two parameters.

Our small sample size is one of this study's major limitations, attributable by 1). Many Parkinson's disease patients were treated with MAO-B inhibitors prior to being recruited and had to be excluded as previous studies have shown that they interfere with [^18^F]SMBT-1 uptake; 2). Some Parkinson's disease patients may present with atypical clinical features or concomitant neurological conditions such as cognitive impairment, which may confound the results in this study and were also excluded. Secondly, we did not evaluate the amyloid burden in our participants, which is one of the known possible confounders for reactive astrocyte activity.

## Conclusion

The automated synthesis of [^18^F]SMBT-1 using the Synthra RNplus module has been successfully established, offering high reproducibility, significant radioactivity yields, radiochemical purity suitable for routine production and further clinical applications without any immediate complication reported after radiotracer administration. Our cohort demonstrated decreased [^18^F]SMBT-1 uptake in patients with moderate severity of Parkinson's disease as they lose the neuroprotective astrocyte function, while severe Parkinson's disease showed increased [^18^F]SMBT-1 uptake, probably due to increased neurotoxic astrocyte activity, resulting in neurodegeneration.

## Data Availability

The original contributions presented in the study are included in the article/Supplementary Material, further inquiries can be directed to the corresponding author.
