# Supplementary material for: Automated radiosynthesis and clinical experience of [18F]SMBT-1 PET imaging for in vivo evaluation of reactive astrocyte in Parkinson's disease: a pilot study
Source: Front Nucl Med. 2025 Dec 8;5:1718255. doi: 10.3389/fnume.2025.1718255 (PMC12719457; doi:10.3389/fnume.2025.1718255)
Supplement: Supplementary file 1 [file Table1.docx]

**Supplemental Information**

**Supplemental Table 1.** Synthesis preparation of the optimal condition for radiosynthesis of [^18^F]SMBT-1via the automated synthesizer module (Synthra RNplus).

| **Vial No. and Holder** | **Reagents and Materials** |
| --- | --- |
| **A1** | 1. mL of Elution Solution   (K_2_CO_3_: 1.5 mg in 0.13 mL of Water + K_222_, 8.0 mg in 0.45 mL of ACN) |
| **A2** | 1.0 mL Acetonitrile |
| **A3** | 2.0 mg of SMBT-1 precursor (THK-5475) in 0.9 mL of DMSO Anh. |
| **A4** | 0.2 mL of 2.0 M HCl |
| **A5** | 8.0 mL of 0.2 M KOAc |
| **A6** | 10.0 mL of Sterile Water for injection |
| **A7** | 1.2 mL of 70% EtOH |
| **B1** | 2.8 mL of Mobile Phase Solution (20 mM NaH_2_PO_4_/acetonitrile, 62/38) |
| **C1** | 4.0 mL of Sodium Ascorbate in 0.9% normal saline solution (NSS)  (5.9 mg Sodium Ascorbate in 10.0 mL of 0.9%NSS) |
| **C2** | 1.5 mL of 70% EtOH |
| **C3** | 15.0 mL of Sterile Water for Injection |
| **SPE Vial** | 45.0 mL of Sterile Water for Injection |
| **Transfer Product Vial** | 4.0 mL of Sodium Ascorbate in 0.9% NSS |
| **Final Product Vial** | 10.5 mL of 0.9% normal saline solution  (passed through Cathivex GV membrane filter 0.22 μm) |
| **F-18 Separation Holder** | Install QMA cartridge between V1 and V13  (Conditioned: 10 mL of 0.05 M K_2_CO_3_ and 20 mL of Water and flush to dry) |
| **Vessel 1 Separation Cartridge Holder** | Install tC18 cartridge-1 between V15 and V16  (Conditioned: 10 mL of EtOH and 20 mL of Water and flush to dry) |
| **SPE Cartridge Holder** | Install tC18 cartridge-2 between V35 and V36  (Conditioned: 10 mL of EtOH and 20 mL of Water and flush to dry) |
| **Dewar Tank** | Fill liquid Nitrogen approximately 2/3 of tank |

**Supplemental Table 2.** Summary of procedure and conditions for radiosynthesis of [^18^F]SMBT-1via the automated synthesizer module (Synthra RNplus)

| **Steps** | **Operation** | **Condition** |
| --- | --- | --- |
| 1 | Transfer of F-18 | Transfer the aqueous F-18 solution from the Cyclotron (> 111 GBq) to the synthesizer module (Receiving F-18 vial). |
| 2 | Trap F-18 on QMA | Transfer the aqueous F-18 solution (~2.8 mL) from receiving F-18 vial passed through the QMA Cartridge to separate the F-18 from water. |
| 3 | Eluting of F-18 from QMA to the Reactor 1 | The elution solution (1.0 mL) from A1 passed through the QMA to the reaction vial 1. |
| 4 | Drying of F-18 | Heat and stir at 100°C, 3 min then Heat up to 120°C, 1 min and cooldown to 80°C (Helium gas flow and vacuum). |
| 5 | Azeotropic drying | Heat and stir at 120°C, 2 min then cooldown to 80°C and to 60°C  (Helium gas flow and vacuum). |
| 6 | Addition of Precursor | Transfer Precursor solution (0.9 mL) from A3 to the reaction vial 1. |
| 7 | Fluorination | Heat and stir at 110°C, 10 min, then cooldown to 50°C |
| 8 | Deprotection | Transfer 2.0 M HCl (0.2 mL) from A4 to the reaction vial 1. Heat and stir at 110°C, 3 min and then cooldown to 50°C. |
| 9 | Reaction Quench | Transfer 0.2 M KOAc (8.0 mL) from A5 to the reaction vial 1 while transfer the solution passed through the tC18(1) cartridge to the waste. |
| 10 | Rinsing the reactor 1 and SPE | Washed the trapped crude product with water for injection (10.0 mL) from A6 to reaction vial 1 then passed through tC18 cartridge (1) to the waste. |
| 11 | Eluting Crude Product from tC18 to Reactor 2 | Transfer 70% EtOH (1.2 mL) from A7 to the reaction vial 1 and then passed through tC18(1) cartridge to reaction vial 2. |
| 12 | Dilution of the Eluate | Transfer mobile phase solution (2.8 mL) from B1 to the reaction vial 2 and then stir. (Mobile Phase: 20 mM NaH_2_PO_4_/acetonitrile, 62/38) |
| 13 | HPLC Separation | Column: Inertsil ODS-4 (10 mm x 250 mm, 5 μm)  Mobile Phase: 20 mM NaH_2_PO_4_/acetonitrile (62/38)  Flow rate 5.0 mL/min, UV wavelength = 254 nm,  Collection: Radioactive peak @16~17 min to the SPE vial |
| 14 | Extraction of the product | - Fraction is diluted with sterile water for injection (45.0 mL) - Load onto tC18 cartridge (2) - Washing with water (15 mL) from C3 to waste - Elution with 70% EtOH (1.5 mL) from C2 to transfer product vial that contained sodium ascorbate in 0.9% NSS (4.0 mL) |
| 15 | Formulation | - Transfer sodium ascorbate in 0.9% NSS (4.0 mL) from C1 pass through tC18 cartridge (2) to transfer product vial. - Transfer all solution (~9.5 mL) from transfer product vial to final product vial, which contained 0.9% NSS (10.5 mL) pass through the sterile membrane filter 0.22 μm (Cathivex GV) |

**Supplemental Table 3.** The amounts of all chemical reagents of each optimization trial and final optimization method.

| **Vials** | **Chemical Reagents** | **Trial 1** | **Trial 2** | **Trial 3** | **Final Trial** |
| --- | --- | --- | --- | --- | --- |
| **A1** | Eluting Solution (K_222_ + K_2_CO_3_) | 0.58 mL | 1.0 mL | 1.0 mL | 1.0 mL |
| **A2** | Acetonitrile | 1.0 mL | 1.0 mL | 1.0 mL | 1.0 mL |
| **A3** | Precursor 2 mg in DMSO anhydrous | 0.45 mL | 0.90 mL | 0.9 mL | 0.9 mL |
| **A4** | 2 M HCl | 0.20 mL | 0.20 mL | 0.2 mL | 0.2 mL |
| **A5** | 0.2 M KOAc | 4.0 mL | 8.0 mL | 8.0 mL | 8.0 mL |
| **A6** | Sterile Water | 10.0 mL | 10.0 mL | 10.0 mL | 10.0 mL |
| **A7** | 70% EtOH | 0.70 mL | 1.0 mL | 1.2 mL | 1.2 mL |
| **B1** | HPLC Mobile phase | 3.0 mL | 3.0 mL | 2.8 mL | 2.8 mL |
| **C1** | Sodium Ascorbate in 0.9% NNS | N.A. | N.A. | N.A. | 4.0 mL |
| **C2** | 70% EtOH | 1.5 mL | 1.5 mL | 1.5 mL | 1.5 mL |
| **C3** | Sterile Water | 15.0 mL | 15.0 mL | 15.0 mL | 15.0 mL |
| **SPE** | Sterile Water | 35.0 mL | 35.0 mL | 45.0 mL | 45.0 mL |
| **Transfer Product Vial** | Sodium Ascorbate in 0.9% NNS | 8.0 mL | 8.0 mL | 8.0 mL | 4.0 mL |
| **Final Product Vial** | 0.9% NNS | 10.5 mL | 10.5 mL | 10.5 mL | 10.5 mL |
| **Radioactivity** | **Start Activity (SOS)** | 73.26 GBq | 48.10 GBq | 36.63 GBq | 118.4 GBq |
|  | **Final Product Activity (EOS)** | 0.43 GBq | 4.94 GBq | 3.07 GBq | 20.20 Gbq |
|  | **Decay Collected Yield (D.C. Yield)** | 0.88 % | 16.09 % | 14.17% | 28.81 % |

**Supplemental Table 4.** The distribution of ¹⁸F-radioactivity remaining on various components of the module of each trial.

| **Trial 1** | **Activity (GBq)** | **Time** | **Elapsed time (min)** | **Decay-Corrected (GBq)** | **D.C. %** |
| --- | --- | --- | --- | --- | --- |
| **Starting F-18** | 73.26 | 13:10 | 0 | - | 100.00 |
| **Final Product vial** | 0.43 | 14:15 | 65 | 0.65 | 0.88 |
| **QMA** | 13.91 | 15:59 | 169 | 40.41 | 55.16 |
| **tC18 cartridge-1** | 5.05 | 16:00 | 170 | 14.76 | 20.15 |
| **tC18 cartridge-2** | 0.017 | 16:01 | 171 | 0.051 | 0.07 |

| **Trial 2** | **Activity (GBq)** | **Time** | **Elapsed time (min)** | **Decay-Corrected (GBq)** | **D.C. %** |
| --- | --- | --- | --- | --- | --- |
| **Starting F-18** | 48.10 | 13:27 | 0 | - | 100.00 |
| **Final Product vial** | 4.94 | 14:38 | 71 | 7.74 | 16.09 |
| **QMA** | 0.84 | 16:44 | 197 | 2.91 | 6.05 |
| **tC18 cartridge-1** | 5.01 | 16:42 | 195 | 17,13 | 35.62 |
| **tC18 cartridge-2** | 0.19 | 16:43 | 196 | 0.67 | 1.39 |

| **Trial 3** | **Activity (GBq)** | **Time** | **Elapsed time (min)** | **Decay-Corrected (GBq)** | **D.C. %** |
| --- | --- | --- | --- | --- | --- |
| **Starting F-18** | 36.63 | 12:32 | 0 | - | 100.00 |
| **Final Product vial** | 3.07 | 13:55 | 83 | 5,19 | 14.17 |
| **QMA** | 0.44 | 16:07 | 215 | 1.71 | 4.68 |
| **tC18 cartridge-1** | 0.31 | 16:08 | 216 | 1.22 | 3.32 |
| **tC18 cartridge-2** | 0.11 | 16:08 | 216 | 0.44 | 1.20 |

**Supplemental Table 5.** The quality control (QC) method and acceptance criterion.

| **Test Conducted at Release** | **Acceptance criterion** |
| --- | --- |
| **Appearance**  Formulation  Clarity | Solution  Colorless to pale yellow and Clear (free of particles) |
| **pH** | 4.5 ≤ pH ≤ 8.5 |
| **Identity**  Radionuclidic identity of ^18^F (gamma spectrometry)  Radionuclidic identity of ^18^F (half-life measurement)  Radiochemical identity of [^18^F]SMBT-1  (Compare to Standard) | 0.511 ± 0.020 MeV (a sum peak of 1.022 ± 0.020 MeV may be observed)  110 ± 5 min half-life time  0.9 ≤ RRT ≤ 1.1 |
| **Purity**  Radionuclidic purity of ^18^F (gamma spectrometry)  Radiochemical purity of [^18^F]SMBT-1 (HPLC) | ≥ 99.5% (US), ≥ 99.9% (EU)  ≥ 90.0% |
| **Chemical concentration**  [^18/19^F]SMBT-1 (HPLC)  Sum of unspecified impurities (HPLC) | ≤ 5.0 μg/mL  ≤ 5.0 μg/mL |
| **Residual solvents** (GC)  Ethanol  Acetonitrile  DMSO | ≤ 10%  ≤ 410 μg/mL  ≤ 5000 μg/mL |
| **Kryptofix content** (TLC) | ≤ 50 μg/mL |
| **Pyrogenicity (BET)** | < 17.5 EU/mL |
| **Sterile filter integrity** (Bubble Point Test) | Bubble point ≥ 50 psi |
| **Sterility Test** | Sterile |
| **Single dose volume not to exceed**  Volume  Total unspecified impurities | ≤ 10.0 mL  ≤ 5.0 μg |

**Supplemental Table 6.** The condition of semi-preparative and analytical HPLC and retention time of SMBT-1 standard.

|  | **Semi-preparative HPLC** | **Analytical HPLC** |
| --- | --- | --- |
| **Stationary Phase**  **(Column)** | Inertsil ODS-4 column, GL Sciences Inc. (10 x 250 mm, 5 μm) | Inertsil ODS-4 column, GL Sciences Inc.  (4.6 x 150 mm, 5 μm) |
| **Mobile Phase** | 20 mM NaH_2_PO_4_/ACN, 62:38 (v/v) | 20 mM NaH_2_PO_4_/ACN, 65:35 (v/v) |
| **Flow rate** | 5.0 mL/min | 1.5 mL/min |
| **Run Time** | Until End of Synthesis | 15 min |
| **SMBT-1 Standard** | Retention time: 16 - 18 min | Retention time: 6 - 8 min |

**Supplemental Table 7.** The linearity of SMBT-1 standard on the analytical HPLC.

| **Concentration**  **(Injection Volume: 20 μL)** | **SMBT-1** | | |
| --- | --- | --- | --- |
|  | **Retention Time** | **Peak area** | **% RSD** |
| **10 μg/mL (ppm)** | 7.10 | 113.416 | 2.989 |
| **10 μg/mL (ppm)** | 7.18 | 108.688 |  |
| **10 μg/mL (ppm)** | 7.18 | 115.188 |  |
| **50 μg/mL (ppm)** | 7.04 | 596.527 | 5.956 |
| **50 μg/mL (ppm)** | 7.06 | 565.317 |  |
| **50 μg/mL (ppm)** | 7.05 | 529.423 |  |
| **125 μg/mL (ppm)** | 7.01 | 1629.177 | 11.612 |
| **125 μg/mL (ppm)** | 7.01 | 1335.782 |  |
| **125 μg/mL (ppm)** | 7.02 | 1344.925 |  |
| **250 μg/mL (ppm)** | 7.01 | 2553.685 | 3.268 |
| **250 μg/mL (ppm)** | 7.03 | 2409.414 |  |
| **250 μg/mL (ppm)** | 7.00 | 2548.435 |  |
| **500 μg/mL (ppm)** | 6.96 | 5140.118 | 8.049 |
| **500 μg/mL (ppm)** | 6.98 | 4679.253 |  |
| **500 μg/mL (ppm)** | 7.00 | 5499.133 |  |
| **Correlation coefficient** | | 0.9986 | |
| **Slope** | | 10.070 | |
| **Intercept** | | 61.409 | |

**Supplemental Table 8.** The analytical parameters of gas chromatography (GC) for evaluation ethanol content and residue solvents.

| **Oven** | |
| --- | --- |
| Initial temp.: 35°C (On) | Maximum temp.: 250°C |
| 35°C, hold for 5 min  Rate: 60°C /min, Final temp.: 190°C, hold for 3 min | |
| Run time: 10-11 min | |
| **Inlet (Split/Splitless)** | **Detector (FID)** |
| Mode: Split | Temperature: 250°C (On) |
| Initial temp.: 250°C (On) | Hydrogen flow: 30.0 mL/min (On) |
| Split ratio: 20:1 | Air flow: 400.0 mL/min (On) |
| Gas type: Helium | Mode: Constant column + makeup flow |
|  | Combined flow: 40 mL/min |
|  | Makeup flow: On |
|  | Gas Type: Nitrogen |
|  | Flame: On |
|  | Electrometer: On |
|  | Lit offset: 2.0 |
| **Column** | **Signal** |
| Model Number: J&W 19091N-133E HP-INNOWAX | Data rate: 20 Hz |
| Agilent Technologies, 30 m, DI 0.25 mm, film 0.25 µm | |
| Mode: constant flow | |
| Column flow: 4.9 mL/min | |
| **Injector (manual injection)** | |
| Injection Syringe Size: 5.0 µL | |
| Injection volume: 1.0 µL | |

**Supplemental Table 9.**  The linearity test of ethanol content and residue solvents analyzed by gas chromatography (GC).

| **Solution** | **Ethanol** | | | **Acetonitrile** | | | **DMSO** | | |
| --- | --- | --- | --- | --- | --- | --- | --- | --- | --- |
|  | **Conc.**  **(%v/v)** | **Peak area** | **% RSD** | **Conc.**  **(µg /ml)** | **peak area** | **% RSD** | **Conc.**  **(µg /ml)** | **Peak area** | **% RSD** |
| **1%** | 0.0999 | 484.137 | 1.063 | 4.0833 | 2.227 | 1.069 | 50.6833 | 24.769 | 6.154 |
| **1%** | 0.0999 | 471.834 |  | 4.0833 | 2.173 |  | 50.6833 | 22.012 |  |
| **1%** | 0.0999 | 476.411 |  | 4.0833 | 2.183 |  | 50.6833 | 21.614 |  |
| **2%** | 0.1998 | 957.343 | 2.122 | 8.1667 | 4.555 | 1.400 | 101.3667 | 41.044 | 0.316 |
| **2%** | 0.1998 | 1004.53 |  | 8.1667 | 4.665 |  | 101.3667 | 41.125 |  |
| **2%** | 0.1998 | 964.862 |  | 8.1667 | 4.513 |  | 101.3667 | 40.819 |  |
| **5%** | 0.4994 | 2383.98 | 4.831 | 20.416 | 11.300 | 4.045 | 253.4167 | 111.475 | 6.615 |
| **5%** | 0.4994 | 2684.00 |  | 20.416 | 12.478 |  | 253.4167 | 129.733 |  |
| **5%** | 0.4994 | 2543.62 |  | 20.416 | 11.893 |  | 253.4167 | 127.492 |  |
| **10%** | 0.9988 | 4864.68 | 1.967 | 40.833 | 23.231 | 0.754 | 506.8333 | 262.714 | 6.408 |
| **10%** | 0.9988 | 4789.83 |  | 40.833 | 22.939 |  | 506.8333 | 227.910 |  |
| **10%** | 0.9988 | 5020.85 |  | 40.833 | 23.356 |  | 506.8333 | 261.186 |  |
| **50%** | 4.9939 | 23254.8 | 1.440 | 204.1667 | 113.714 | 0.751 | 2534.1667 | 1199.19 | 3.181 |
| **50%** | 4.9939 | 23929.2 |  | 204.1667 | 115.158 |  | 2534.1667 | 1287.63 |  |
| **50%** | 4.9939 | 23177.3 |  | 204.1667 | 113.118 |  | 2534.1667 | 1279.62 |  |
| **100%** | 9.9879 | 47017.9 | 3.944 | 408.3333 | 232.38 | 1.090 | 5068.3333 | 2690.67 | 0.967 |
| **100%** | 9.9879 | 45748.1 |  | 408.3333 | 226.449 |  | 5068.3333 | 2688.65 |  |
| **100%** | 9.9879 | 50215.4 |  | 408.3333 | 230.781 |  | 5068.3333 | 2745.18 |  |
| **120%** | 11.985 | 55246.3 | 0.771 | 490.0000 | 272.097 | 0.124 | 6082.0000 | 3223.2 | 2.256 |
| **120%** | 11.985 | 54521.5 |  | 490.0000 | 272.510 |  | 6082.0000 | 3376.95 |  |
| **120%** | 11.985 | 55530.0 |  | 490.0000 | 272.928 |  | 6082.0000 | 3217.63 |  |
| **Correlation coefficient** | 0.9995 | | | 0.9999 | | | 0.9993 | | |
| **Slope** | 4653.9 | | | 0.5583 | | | 0.5387 | | |
| **Intercept** | 175.07 | | | 0.2318 | | | -25.296 | | |

**Supplemental Table 10.** The stability test of 3 validations of [^18^F]SMBT-1

| **Stability Test** | **Validation 1** | | **Validation 2** | | **Validation 3** | |
| --- | --- | --- | --- | --- | --- | --- |
| **Time** | **RT#1 (min)** | **RCP #1** | **RT#2 (min)** | **RCP #2** | **RT#3 (min)** | **RCP #3** |
| **0 hour** | 7.10 | > 99.9 % | 7.00 | >99.9 % | 6.91 | 99.8 % |
| **2 hour** | 7.10 | >99.9 % | 7.00 | >99.9 % | 7.27 | 98.8 % |
| **4 hour** | 7.04 | >99.9 % | 6.18 | >99.9 % | 7.24 | 99.2 % |
| **6 hour** | 6.67 | >99.9 % | 6.79 | >99.9 % | 7.35 | 99.6 % |
| **8 hour** | 7.06 | >99.9 % | 6.63 | >99.9 % | 7.19 | >99.9 % |
| **10 hour** | 7.10 | >99.9 % | 6.91 | > 99.9% | 7.33 | >99.9 % |

**Supplemental Figure 1.** Standard curve of SMBT-1 on analytical HPLC with UV/Vis and Gamma detector

**Supplemental Figure 2.** Standard Curve of ethanol content and residue solvents.

**Supplemental Figure 3.** The radiochromatogram of purificaiton via the semi-preparative HPLC.

**Supplemental Figure 4.** The chromatogram of stability test results of [^18^F]SMBT-1, analysis in every 2 hours after end of synthesis EOS (RCP >95% at each time of analysis).
