# Supplementary material for: Automated radiosynthesis and clinical experience of [18F]SMBT-1 PET imaging for in vivo evaluation of reactive astrocyte in Parkinson's disease: a pilot study
Source: Front Nucl Med. 2025 Dec 8;5:1718255. doi: 10.3389/fnume.2025.1718255 (PMC12719457; doi:10.3389/fnume.2025.1718255)
Supplement: Supplementary file 2 [file Datasheet1.pdf]

Supplemental Information

Supplemental Table 1. Synthesis preparation of the optimal condition for radiosynthesis of [^{18}F]SMBT-1via the automated synthesizer module (Synthra RNplus).

Vial No. and Holder	Reagents and Materials
**A1**	1.0 mL of Elution Solution<br>(K_2CO_3: 1.5 mg in 0.13 mL of Water + K_{222}, 8.0 mg in 0.45 mL of ACN)
**A2**	1.0 mL Acetonitrile
**A3**	2.0 mg of SMBT-1 precursor (THK-5475) in 0.9 mL of DMSO Anh.
**A4**	0.2 mL of 2.0 M HCl
**A5**	8.0 mL of 0.2 M KOAc
**A6**	10.0 mL of Sterile Water for injection
**A7**	1.2 mL of 70% EtOH
**B1**	2.8 mL of Mobile Phase Solution (20 mM NaH_2PO_4/acetonitrile, 62/38)
**C1**	4.0 mL of Sodium Ascorbate in 0.9% normal saline solution (NSS)<br>(5.9 mg Sodium Ascorbate in 10.0 mL of 0.9%NSS)
**C2**	1.5 mL of 70% EtOH
**C3**	15.0 mL of Sterile Water for Injection
**SPE Vial**	45.0 mL of Sterile Water for Injection
**Transfer Product Vial**	4.0 mL of Sodium Ascorbate in 0.9% NSS
**Final Product Vial**	10.5 mL of 0.9% normal saline solution<br>(passed through Cathivex GV membrane filter 0.22 μm)
**F-18 Separation Holder**	Install QMA cartridge between V1 and V13<br>(Conditioned: 10 mL of 0.05 M K_2CO_3 and 20 mL of Water and flush to dry)
**Vessel 1 Separation Cartridge Holder**	Install tC18 cartridge-1 between V15 and V16<br>(Conditioned: 10 mL of EtOH and 20 mL of Water and flush to dry)
**SPE Cartridge Holder**	Install tC18 cartridge-2 between V35 and V36<br>(Conditioned: 10 mL of EtOH and 20 mL of Water and flush to dry)
**Dewar Tank**	Fill liquid Nitrogen approximately 2/3 of tank

Supplemental Table 2. Summary of procedure and conditions for radiosynthesis of [^{18}F]SMBT-1via the automated synthesizer module (Synthra RNplus)

Steps	Operation	Condition
1	Transfer of F-18	Transfer the aqueous F-18 solution from the Cyclotron (> 111 GBq) to the synthesizer module (Receiving F-18 vial).
2	Trap F-18 on QMA	Transfer the aqueous F-18 solution (~2.8 mL) from receiving F-18 vial passed through the QMA Cartridge to separate the F-18 from water.
3	Eluting of F-18 from QMA to the Reactor 1	The elution solution (1.0 mL) from A1 passed through the QMA to the reaction vial 1.
4	Drying of F-18	Heat and stir at 100°C, 3 min then Heat up to 120°C, 1 min and cooldown to 80°C (Helium gas flow and vacuum).
5	Azeotropic drying	Heat and stir at 120°C, 2 min then cooldown to 80°C and to 60°C (Helium gas flow and vacuum).
6	Addition of Precursor	Transfer Precursor solution (0.9 mL) from A3 to the reaction vial 1.
7	Fluorination	Heat and stir at 110°C, 10 min, then cooldown to 50°C
8	Deprotection	Transfer 2.0 M HCl (0.2 mL) from A4 to the reaction vial 1. Heat and stir at 110°C, 3 min and then cooldown to 50°C.
9	Reaction Quench	Transfer 0.2 M KOAc (8.0 mL) from A5 to the reaction vial 1 while transfer the solution passed through the tC18(1) cartridge to the waste.
10	Rinsing the reactor 1 and SPE	Washed the trapped crude product with water for injection (10.0 mL) from A6 to reaction vial 1 then passed through tC18 cartridge (1) to the waste.
11	Eluting Crude Product from tC18 to Reactor 2	Transfer 70% EtOH (1.2 mL) from A7 to the reaction vial 1 and then passed through tC18(1) cartridge to reaction vial 2.
12	Dilution of the Eluate	Transfer mobile phase solution (2.8 mL) from B1 to the reaction vial 2 and then stir. (Mobile Phase: 20 mM NaH_2PO_4/acetonitrile, 62/38)
13	HPLC Separation	Column: Inertsil ODS-4 (10 mm x 250 mm, 5 μm)<br>Mobile Phase: 20 mM NaH_2PO_4/acetonitrile (62/38)<br>Flow rate 5.0 mL/min, UV wavelength = 254 nm,<br>Collection: Radioactive peak @16~17 min to the SPE vial
14	Extraction of the product	• Fraction is diluted with sterile water for injection (45.0 mL)<br>• Load onto tC18 cartridge (2)<br>• Washing with water (15 mL) from C3 to waste<br>• Elution with 70% EtOH (1.5 mL) from C2 to transfer product vial that contained sodium ascorbate in 0.9% NSS (4.0 mL)
15	Formulation	• Transfer sodium ascorbate in 0.9% NSS (4.0 mL) from C1 pass through tC18 cartridge (2) to transfer product vial.<br>• Transfer all solution (~9.5 mL) from transfer product vial to final product vial, which contained 0.9% NSS (10.5 mL) pass through the sterile membrane filter 0.22 μm (Cathivex GV)

Supplemental Table 3. The amounts of all chemical reagents of each optimization trial and final optimization method.

Vials	Chemical Reagents	Trial 1	Trial 2	Trial 3	Final Trial
**A1**	Eluting Solution (K_{222} + K_2CO_3)	0.58 mL	1.0 mL	1.0 mL	1.0 mL
**A2**	Acetonitrile	1.0 mL	1.0 mL	1.0 mL	1.0 mL
**A3**	Precursor 2 mg in DMSO anhydrous	0.45 mL	0.90 mL	0.9 mL	0.9 mL
**A4**	2 M HCl	0.20 mL	0.20 mL	0.2 mL	0.2 mL
**A5**	0.2 M KOAc	4.0 mL	8.0 mL	8.0 mL	8.0 mL
**A6**	Sterile Water	10.0 mL	10.0 mL	10.0 mL	10.0 mL
**A7**	70% EtOH	0.70 mL	1.0 mL	1.2 mL	1.2 mL
**B1**	HPLC Mobile phase	3.0 mL	3.0 mL	2.8 mL	2.8 mL
**C1**	Sodium Ascorbate in 0.9% NNS	N.A.	N.A.	N.A.	4.0 mL
**C2**	70% EtOH	1.5 mL	1.5 mL	1.5 mL	1.5 mL
**C3**	Sterile Water	15.0 mL	15.0 mL	15.0 mL	15.0 mL
**SPE**	Sterile Water	35.0 mL	35.0 mL	45.0 mL	45.0 mL
**Transfer Product Vial**	Sodium Ascorbate in 0.9% NNS	8.0 mL	8.0 mL	8.0 mL	4.0 mL
**Final Product Vial**	0.9% NNS	10.5 mL	10.5 mL	10.5 mL	10.5 mL
**Radioactivity**	**Start Activity (SOS)**	73.26 GBq	48.10 GBq	36.63 GBq	118.4 GBq
	**Final Product Activity (EOS)**	0.43 GBq	4.94 GBq	3.07 GBq	20.20 Gbq
	**Decay Collected Yield (D.C. Yield)**	0.88 %	16.09 %	14.17%	28.81 %

Supplemental Table 4. The distribution of ^{18}F-radioactivity remaining on various components of the module of each trial.

Trial 1	Activity (GBq)	Time	Elapsed time (min)	Decay-Corrected (GBq)	D.C. %
**Starting F-18**	73.26	13:10	0	-	100.00
**Final Product vial**	0.43	14:15	65	0.65	0.88
**QMA**	13.91	15:59	169	40.41	55.16
**tC18 cartridge-1**	5.05	16:00	170	14.76	20.15
**tC18 cartridge-2**	0.017	16:01	171	0.051	0.07

Trial 2	Activity (GBq)	Time	Elapsed time (min)	Decay-Corrected (GBq)	D.C. %
**Starting F-18**	48.10	13:27	0	-	100.00
**Final Product vial**	4.94	14:38	71	7.74	16.09
**QMA**	0.84	16:44	197	2.91	6.05
**tC18 cartridge-1**	5.01	16:42	195	17,13	35.62
**tC18 cartridge-2**	0.19	16:43	196	0.67	1.39

Trial 3	Activity (GBq)	Time	Elapsed time (min)	Decay-Corrected (GBq)	D.C. %
**Starting F-18**	36.63	12:32	0	-	100.00
**Final Product vial**	3.07	13:55	83	5,19	14.17
**QMA**	0.44	16:07	215	1.71	4.68
**tC18 cartridge-1**	0.31	16:08	216	1.22	3.32
**tC18 cartridge-2**	0.11	16:08	216	0.44	1.20

Supplemental Table 5. The quality control (QC) method and acceptance criterion.

Test Conducted at Release	Acceptance criterion
**Appearance**<br>Formulation<br>Clarity	 Solution<br>Colorless to pale yellow and Clear (free of particles)
**pH**	$4.5 \leq pH \leq 8.5$
**Identity**<br>Radionuclidic identity of ^{18}F (gamma spectrometry)<br><br><br>Radionuclidic identity of ^{18}F (half-life measurement)<br>Radiochemical identity of [^{18}F]SMBT-1<br>(Compare to Standard)	 0.511 ± 0.020 MeV (a sum peak of 1.022 ± 0.020 MeV may be observed)<br><br>110 ± 5 min half-life time<br>0.9 ≤ RRT ≤ 1.1
**Purity**<br>Radionuclidic purity of ^{18}F (gamma spectrometry)<br>Radiochemical purity of [^{18}F]SMBT-1 (HPLC)	 ≥ 99.5% (US), ≥ 99.9% (EU)<br>≥ 90.0%
**Chemical concentration**<br>[$^{18/19}F$]SMBT-1 (HPLC)<br>Sum of unspecified impurities (HPLC)	 ≤ 5.0 µg/mL<br>≤ 5.0 µg/mL
**Residual solvents** (GC)<br>Ethanol<br>Acetonitrile<br>DMSO	 ≤ 10%<br>≤ 410 µg/mL<br>≤ 5000 µg/mL
**Kryptofix content** (TLC)	≤ 50 µg/mL
**Pyrogenicity (BET)**	< 17.5 EU/mL
**Sterile filter integrity** (Bubble Point Test)	Bubble point ≥ 50 psi
**Sterility Test**	Sterile
**Single dose volume not to exceed**<br>Volume<br>Total unspecified impurities	 ≤ 10.0 mL<br>≤ 5.0 µg

Supplemental Table 6. The condition of semi-preparative and analytical HPLC and retention time of SMBT-1 standard.

	**Semi-preparative HPLC**	**Analytical HPLC**
**Stationary Phase (Column)**	Inertsil ODS-4 column, GL Sciences Inc. (10 x 250 mm, 5 μm)	Inertsil ODS-4 column, GL Sciences Inc. (4.6 x 150 mm, 5 μm)
**Mobile Phase**	20 mM NaH_2PO_4/ACN, 62:38 (v/v)	20 mM NaH_2PO_4/ACN, 65:35 (v/v)
**Flow rate**	5.0 mL/min	1.5 mL/min
**Run Time**	Until End of Synthesis	15 min
**SMBT-1 Standard**	Retention time: 16 - 18 min	Retention time: 6 - 8 min

Supplemental Table 7. The linearity of SMBT-1 standard on the analytical HPLC.

Concentration (Injection Volume: 20 μL)	SMBT-1		
	Retention Time	Peak area	% RSD
**10 μg/mL (ppm)**	7.10	113.416	2.989
**10 μg/mL (ppm)**	7.18	108.688	
**10 μg/mL (ppm)**	7.18	115.188	
**50 μg/mL (ppm)**	7.04	596.527	5.956
**50 μg/mL (ppm)**	7.06	565.317	
**50 μg/mL (ppm)**	7.05	529.423	
**125 μg/mL (ppm)**	7.01	1629.177	11.612
**125 μg/mL (ppm)**	7.01	1335.782	
**125 μg/mL (ppm)**	7.02	1344.925	
**250 μg/mL (ppm)**	7.01	2553.685	3.268
**250 μg/mL (ppm)**	7.03	2409.414	
**250 μg/mL (ppm)**	7.00	2548.435	
**500 μg/mL (ppm)**	6.96	5140.118	8.049
**500 μg/mL (ppm)**	6.98	4679.253	
**500 μg/mL (ppm)**	7.00	5499.133	
**Correlation coefficient**		0.9986	
**Slope**		10.070	
**Intercept**		61.409	

Supplemental Table 8. The analytical parameters of gas chromatography (GC) for evaluation ethanol content and residue solvents.

**Oven**	
Initial temp.: 35°C (On)	Maximum temp.: 250°C
35°C, hold for 5 min<br>Rate: 60°C /min, Final temp.: 190°C, hold for 3 min	
Run time: 10-11 min	
**Inlet (Split/Splitless)**	**Detector (FID)**
Mode: Split	Temperature: 250°C (On)
Initial temp.: 250°C (On)	Hydrogen flow: 30.0 mL/min (On)
Split ratio: 20:1	Air flow: 400.0 mL/min (On)
Gas type: Helium	Mode: Constant column + makeup flow
	Combined flow: 40 mL/min
	Makeup flow: On
	Gas Type: Nitrogen
	Flame: On
	Electrometer: On
	Lit offset: 2.0
**Column**	**Signal**
Model Number: J&W 19091N-133E HP-INNOWAX	Data rate: 20 Hz
Agilent Technologies, 30 m, DI 0.25 mm, film 0.25 µm	
Mode: constant flow	
Column flow: 4.9 mL/min	
**Injector (manual injection)**	
Injection Syringe Size: 5.0 µL	
Injection volume: 1.0 µL	

Supplemental Table 9. The linearity test of ethanol content and residue solvents analyzed by gas chromatography (GC).

Solution	Ethanol			Acetonitrile			DMSO		
	Conc. (%v/v)	Peak area	% RSD	Conc. (µg /ml)	peak area	% RSD	Conc. (µg /ml)	Peak area	% RSD
1%	0.0999	484.137	1.063	4.0833	2.227	1.069	50.6833	24.769	6.154
1%	0.0999	471.834		4.0833	2.173		50.6833	22.012	
1%	0.0999	476.411		4.0833	2.183		50.6833	21.614	
2%	0.1998	957.343	2.122	8.1667	4.555	1.400	101.3667	41.044	0.316
2%	0.1998	1004.53		8.1667	4.665		101.3667	41.125	
2%	0.1998	964.862		8.1667	4.513		101.3667	40.819	
5%	0.4994	2383.98	4.831	20.416	11.300	4.045	253.4167	111.475	6.615
5%	0.4994	2684.00		20.416	12.478		253.4167	129.733	
5%	0.4994	2543.62		20.416	11.893		253.4167	127.492	
10%	0.9988	4864.68	1.967	40.833	23.231	0.754	506.8333	262.714	6.408
10%	0.9988	4789.83		40.833	22.939		506.8333	227.910	
10%	0.9988	5020.85		40.833	23.356		506.8333	261.186	
50%	4.9939	23254.8	1.440	204.1667	113.714	0.751	2534.1667	1199.19	3.181
50%	4.9939	23929.2		204.1667	115.158		2534.1667	1287.63	
50%	4.9939	23177.3		204.1667	113.118		2534.1667	1279.62	
100%	9.9879	47017.9	3.944	408.3333	232.38	1.090	5068.3333	2690.67	0.967
100%	9.9879	45748.1		408.3333	226.449		5068.3333	2688.65	
100%	9.9879	50215.4		408.3333	230.781		5068.3333	2745.18	
120%	11.985	55246.3	0.771	490.0000	272.097	0.124	6082.0000	3223.2	2.256
120%	11.985	54521.5		490.0000	272.510		6082.0000	3376.95	
120%	11.985	55530.0		490.0000	272.928		6082.0000	3217.63	
**Correlation coefficient**	0.9995			0.9999			0.9993		
**Slope**	4653.9			0.5583			0.5387		
**Intercept**	175.07			0.2318			-25.296		

Supplemental Table 10. The stability test of 3 validations of $[^{18}F]$SMBT-1

**Stability Test**	**Validation 1**		**Validation 2**		**Validation 3**	
**Time**	**RT#1 (min)**	**RCP #1**	**RT#2 (min)**	**RCP #2**	**RT#3 (min)**	**RCP #3**
**0 hour**	7.10	> 99.9 %	7.00	>99.9 %	6.91	99.8 %
**2 hour**	7.10	>99.9 %	7.00	>99.9 %	7.27	98.8 %
**4 hour**	7.04	>99.9 %	6.18	>99.9 %	7.24	99.2 %
**6 hour**	6.67	>99.9 %	6.79	>99.9 %	7.35	99.6 %
**8 hour**	7.06	>99.9 %	6.63	>99.9 %	7.19	>99.9 %
**10 hour**	7.10	>99.9 %	6.91	> 99.9%	7.33	>99.9 %

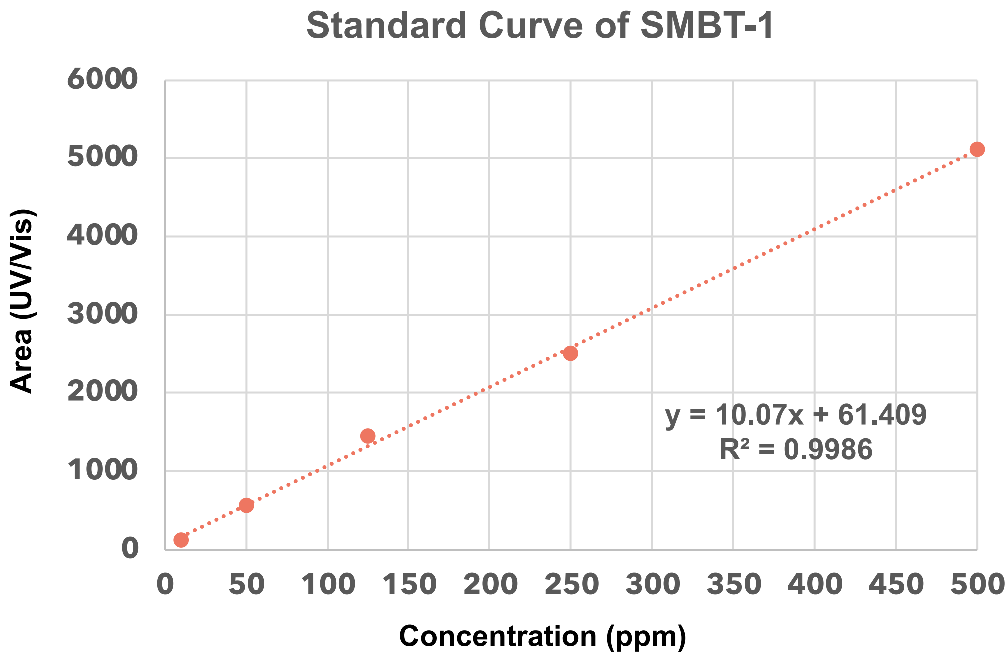

Supplemental Figure 1. Standard curve of SMBT-1 on analytical HPLC with UV/Vis and Gamma detector

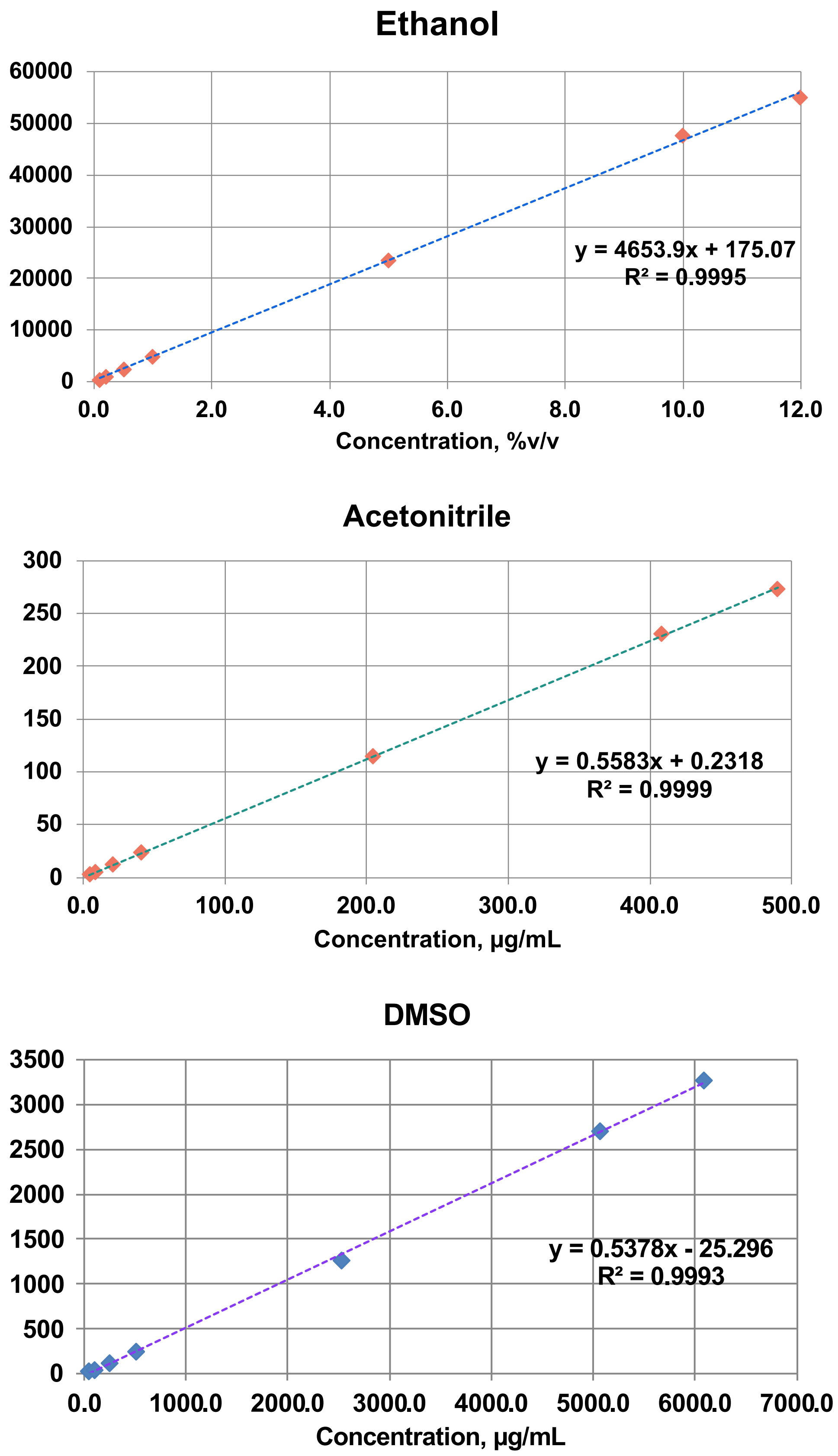

Supplemental Figure 2. Standard Curve of ethanol content and residue solvents.

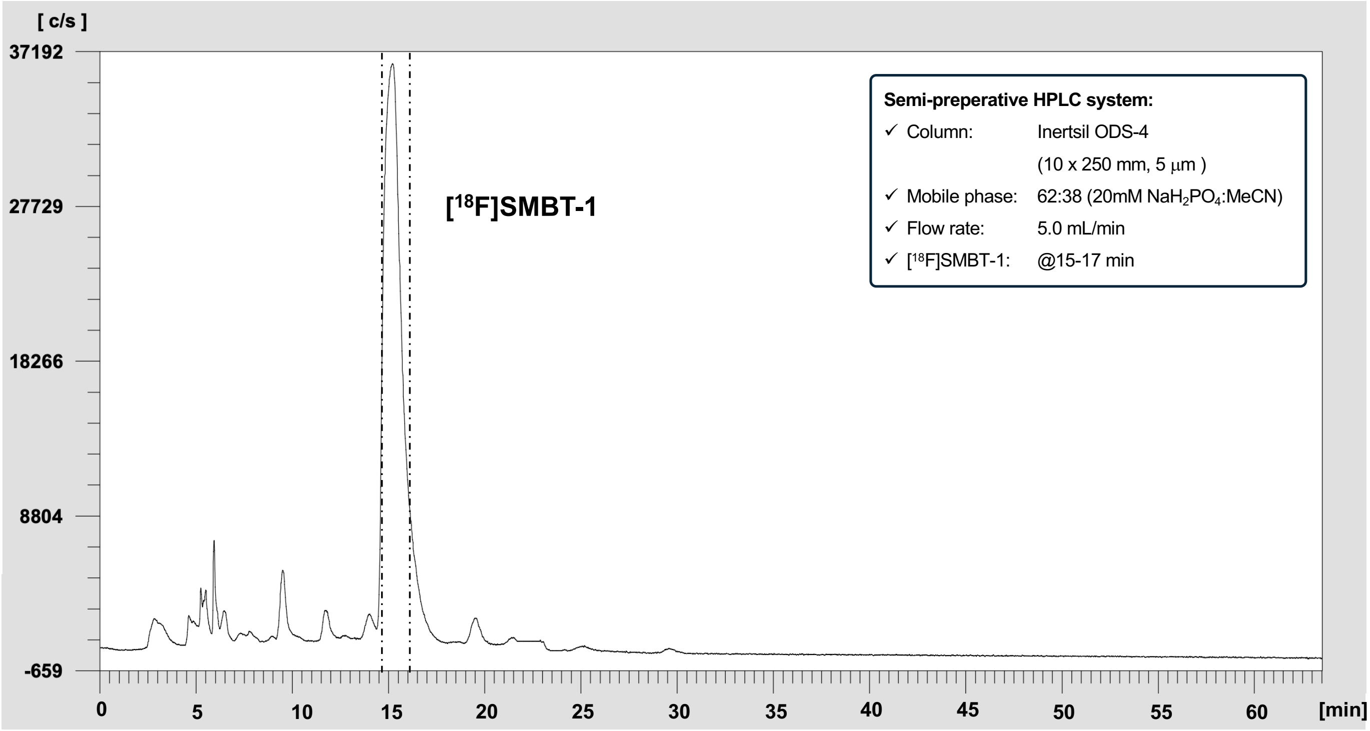

Supplemental Figure 3. The radiochromatogram of purificaiton via the semi-preparative HPLC.

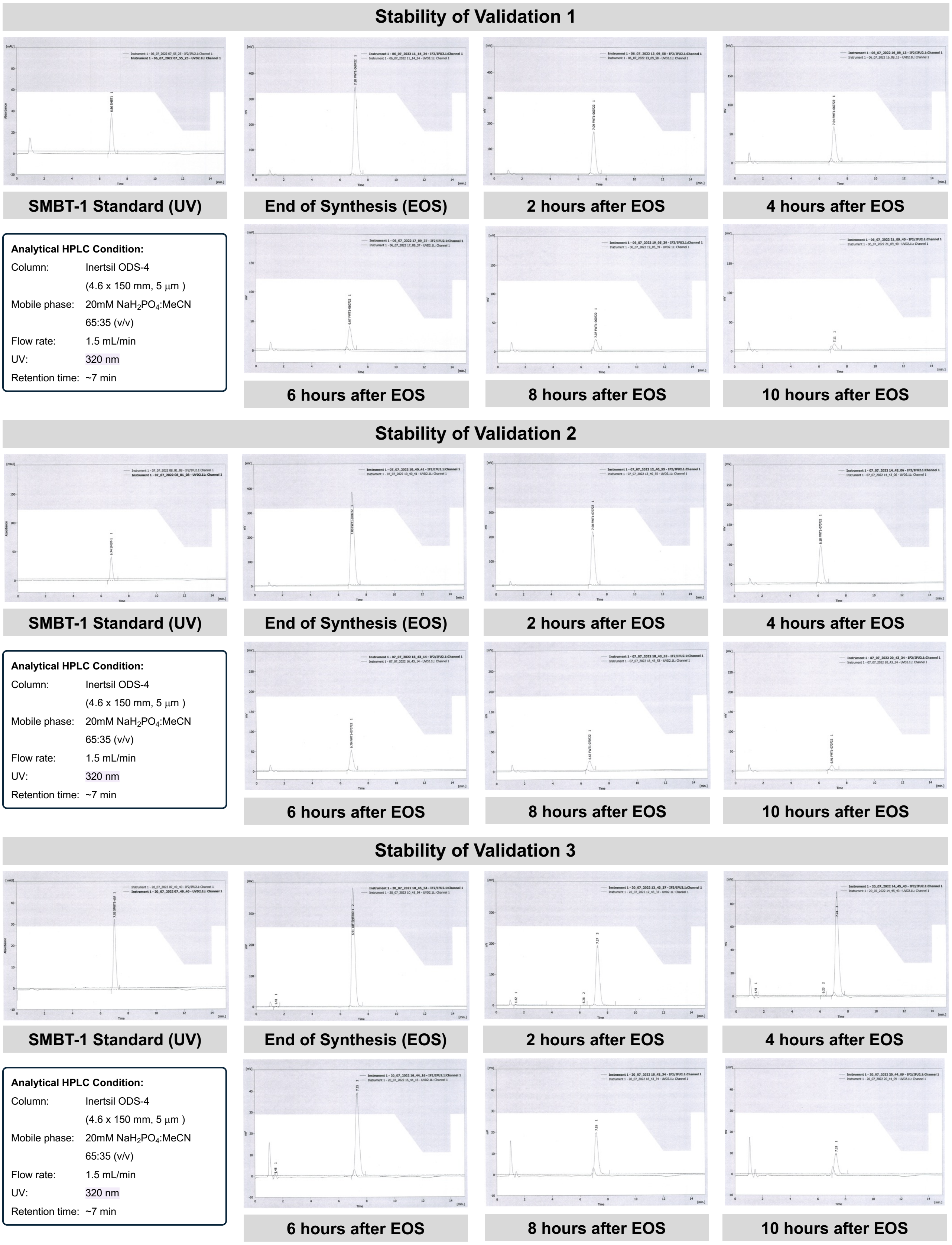

Supplemental Figure 4. The chromatogram of stability test results of [^{18}F]SMBT-1, analysis in every 2 hours after end of synthesis EOS (RCP >95% at each time of analysis).
