# Supplementary figures and images for: Automated radiosynthesis and clinical experience of [18F]SMBT-1 PET imaging for in vivo evaluation of reactive astrocyte in Parkinson's disease: a pilot study
Source: Front Nucl Med. 2025 Dec 8;5:1718255. doi: 10.3389/fnume.2025.1718255 (PMC12719457; doi:10.3389/fnume.2025.1718255)

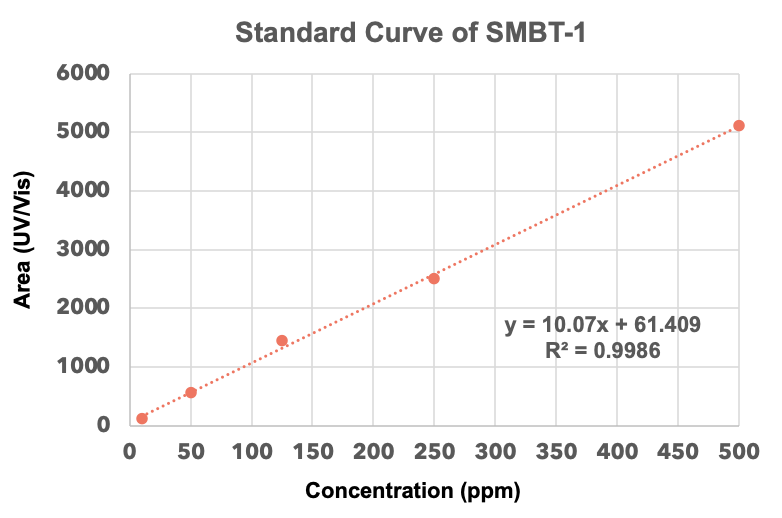

Supplement: Supplementary file 3 [file Image1.tiff]

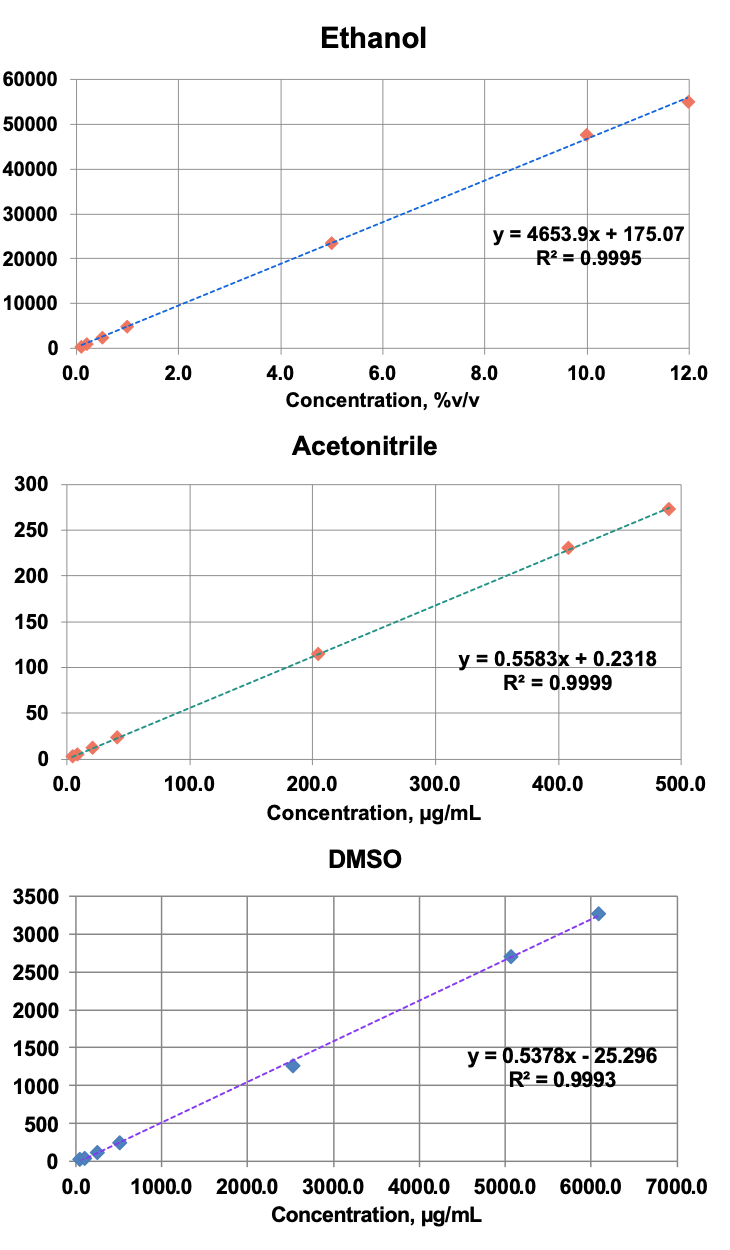

Supplement: Supplementary file 4 [file Image2.tiff]

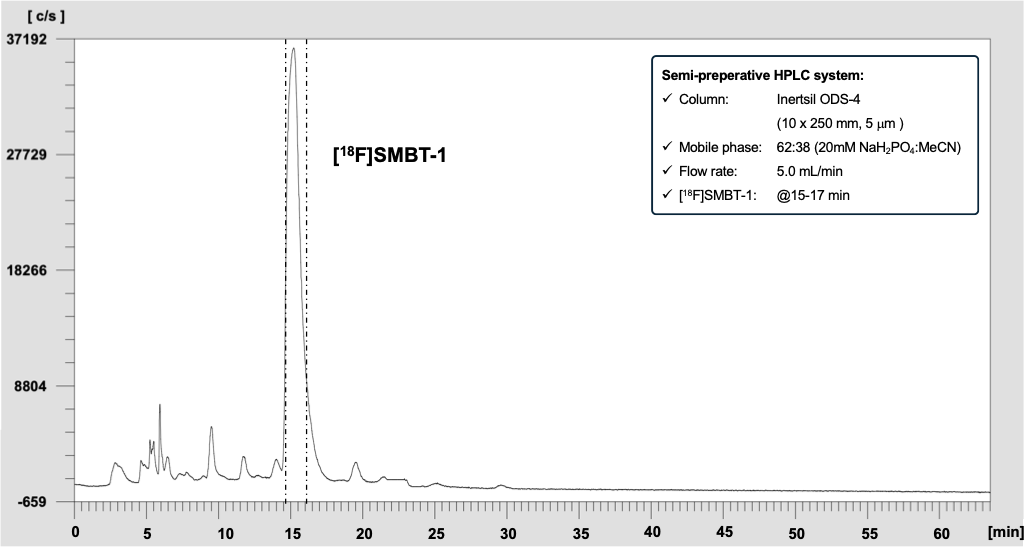

Supplement: Supplementary file 5 [file Image3.tiff]

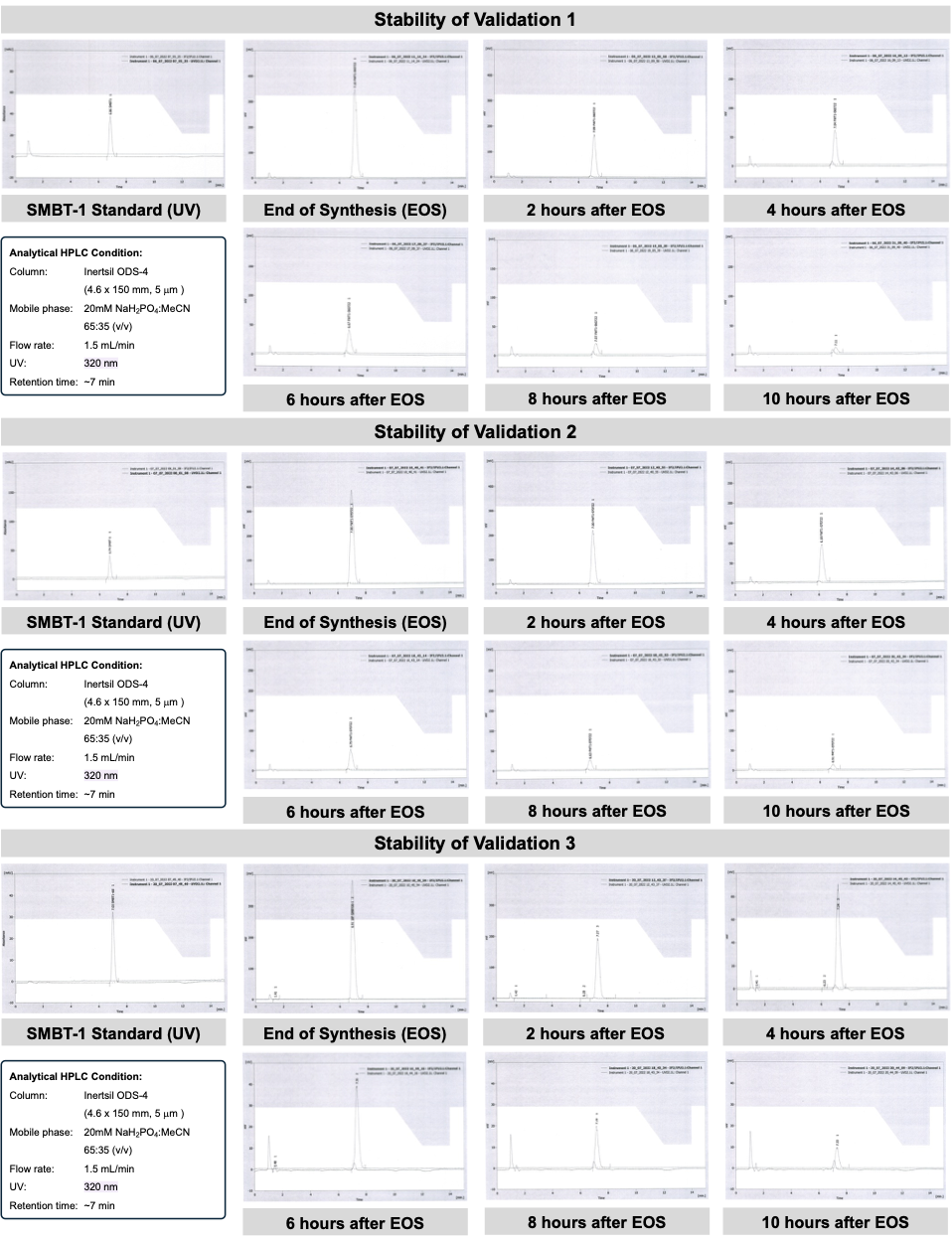

Supplement: Supplementary file 6 [file Image4.tiff]
